# Design decisions and data completeness for experience sampling methods used in psychosis: systematic review

**DOI:** 10.1186/s12888-022-04319-x

**Published:** 2022-10-28

**Authors:** Emilia Deakin, Fiona Ng, Emma Young, Naomi Thorpe, Christopher Newby, Carol Coupland, Michael Craven, Mike Slade

**Affiliations:** 1 School of Health Sciences, Institute of Mental Health, University of Nottingham, Triumph Road, Nottingham, NG7 2TU UK; 2 Nottingham Biomedical Research Centre, University of Nottingham, Nottingham, UK; 3 Nottinghamshire Healthcare NHS Foundation Trust, Nottingham, UK; 4 School of Medicine, University of Nottingham, Nottingham, UK; 5 Human Factors Research Group, Faculty of Engineering, University of Nottingham, Nottingham, UK; 6 Institute of Mental Health, NIHR MindTech MedTech Co-Operative, University of Nottingham, Nottingham, UK; 7 Nord University, Postboks 474, 7801 Namsos, Norway

**Keywords:** Experience sampling methods, ESM, Ecological momentary assessment, EMA, Psychosis, Smartphone

## Abstract

**Background:**

The experience sampling method (ESM) is an intensive longitudinal research method.

Participants complete questionnaires at multiple times about their current or very recent state. The design of ESM studies is complex. People with psychosis have been shown to be less adherent to ESM study protocols than the general population. It is not known how to design studies that increase adherence to study protocols. A lack of typology makes it is hard for researchers to decide how to collect data in a way that allows for methodological rigour, quality of reporting, and the ability to synthesise findings. The aims of this systematic review were to characterise the design choices made in ESM studies monitoring the daily lives of people with psychosis, and to synthesise evidence relating the data completeness to different design choices.

**Methods:**

A systematic review was conducted of published literature on studies using ESM with people with psychosis. Studies were included if they used digital technology for data collection and reported the completeness of the data set. The constant comparative method was used to identify design decisions, using inductive identification of design decisions with simultaneous comparison of design decisions observed. Weighted regression was used to identify design decisions that predicted data completeness. The review was pre-registered (PROSPERO CRD42019125545).

**Results:**

Thirty-eight studies were included. A typology of design choices used in ESM studies was developed, which comprised three superordinate categories of design choice: Study context, ESM approach and ESM implementation. Design decisions that predict data completeness include type of ESM protocol used, length of time participants are enrolled in the study, and if there is contact with the research team during data collection.

**Conclusions:**

This review identified a range of design decisions used in studies using ESM in the context of psychosis. Design decisions that influence data completeness were identified. Findings will help the design and reporting of future ESM studies. Results are presented with the focus on psychosis, but the findings can be applied across different mental health populations.

**Supplementary Information:**

The online version contains supplementary material available at 10.1186/s12888-022-04319-x.

## Background

The experience sampling method (ESM) is an intensive longitudinal research method [1]. ESM is conducted in real world settings as a participant goes about their daily life [2]. Participants complete self-report questions about transient experiences at multiple times, typically followed by questions relating to current environment or context [3]. Prior to the advent of digital technologies, ESM involved filling in a diary or booklet [4]. Most ESM designs are now computerised and allow researchers to identify the exact time a momentary assessment was completed [5]. The review will focus specifically on digital ESM because paper-based approaches are increasingly redundant and the review focus on data completeness is likely to be strongly influenced by data collection approach.

ESM can provide an accurate assessment of phenomena as they occur [2]. It allows researchers to gain more ecologically valid insights into the impact of daily events on participants, which are difficult to measure under laboratory conditions [6]. ESM can be used to examine temporal precedence between variables [2]. By asking participants to report experiences over a period of time, researchers can investigate fluctuations between variables which may not be captured using other methods [7].

ESM has been used widely in mental health research [8], and a review of its use has identified a number of applications including improving understanding of symptoms and social interactions, identifying causes of symptom variation and evaluating treatments [9]. ESM is a valid approach when capturing mental health states in participants with psychosis [10].

Data completeness is a particular challenge in ESM. Missing data is common in research using ESM methods [11]. Data incompleteness can occur for a number of reasons, such as participants finding ESM burdensome and time consuming [2], leading to reduced adherence to the study protocol, resulting in reduced data quantity [12] and quality [13]. Incomplete data sets can cause important aspects of experience to be overlooked by researchers and also bias statistical models used for analysis [14]. People with psychosis have been shown to be less adherent to ESM study protocols than the general population [13]. Studies that recruit people with psychosis have higher rates of participant withdrawal, resulting in fewer participants included in final analyses [15].

### ESM design

Conducting an ESM study involves making several design decisions [16]. For example, deciding when and how frequently participants answer questionnaires. A questionnaire prompt may be sent to participants at pre-defined intervals (time contingent protocol), scheduled at random times (signal contingent protocol) or carried out when a predefined event has occurred (event contingent protocol) [17]. Studies can also use hybrid designs, which combine sampling protocols [9]. Setting the frequency of questionnaire prompts involves consideration of participant burden as well as how rapidly the target phenomenon is expected to vary [4].

There is evidence that design decisions influence completion rates. For example, longer questionnaires have been associated with higher levels of participant burden [18]. Protocol adherence has been shown to reduce over time, and also to be dependent on the time of day a questionnaire is received [13]. A systematic review investigating compliance with study protocols and retention in ESM studies in participants with severe mental illness found that frequent assessments and short intervals between questionnaires reduce data completeness, and increasing participant reimbursement increases data completeness [15].

There is a need for greater consistency in the design of ESM studies [9]. ESM is a collection of methods and is usually reported in relation to general characteristics rather than a defined set of design options [4]. When designing an ESM study, researchers have insufficient evidence on which to base design decisions [18]. Designs of ESM studies are often based on individual research questions [16], leading to a large heterogeneity of designs [15]. Additional methodological research is needed in order for studies to be replicable and standardised [9].

Developing consistency in design is impeded by the absence of a typology of design decisions. No typology for ESM design choices currently exists. A typology could help to define and classify ESM research methods [19], increasing both methodological rigour in developing and reporting individual studies and the ability to compare or combine findings.

### Review aims

The aim of this systematic review is to characterise the design choices made in digital ESM studies monitoring the daily lives of people with psychosis. The objectives in relation to ESM studies involving people with psychosis are:

- (1) to develop a typology of design choices used in digital ESM studies and
- (2) to synthesise evidence relating data completeness to different ESM design choices.

## Methods

A systematic review of the literature was carried out following PRISMA guidance [20]. Studies published in academic journals that met inclusion criteria were assessed for methodological quality. The constant comparative method [21] was used to identify design decisions to produce a typology. Weighted regression was used to identify design decisions that predicted data completeness.

### Eligibility criteria

Inclusion criteria:

• Participants: papers that reported on participants with a clinical or research diagnosis of psychosis, either as a category or by specific diagnosis, e.g., schizophrenia either as the study population or as a separately reported and disaggregable sub-group
• Methods: Studies using ESM to monitor participants with psychosis
• Studies which used digital technology to administer ESM
• Studies which included experience sampling as part of a wider design, e.g. as part of an intervention
• English language full text articles, reviews and conference abstracts
• Papers published from January 2009 to July 2021
• Studies which either reported the completeness of data or gave sufficient data to allow calculation of data completeness where not specifically reported

Exclusion criteria:

• Studies recruiting non clinically diagnosed adults, i.e. participants self-reporting psychosis without clinical or research validation
• Studies using non-digital approaches to data collection
• Lifelogging, quantified self and other self-tracking approaches used by individuals to record personal data, since these are not research methodologies used to collect data for scientific purposes

### Data sources and search strategy

A systematic search was developed and conducted in collaboration with two information specialists with expertise in systematic review searches (authors EY and NT). These data sources and associated search strategy are described below.

Six sources were used.

First, the following electronic databases were searched with a date limit of January 2009 to July 2021 (date of last search): Medline, Embase, PsycInfo (all via Ovid), Cochrane Library, and Web of Science Core Collection. The search terms are described in detail in additional file 1.

Second, the table of contents for the following journals were hand searched: Journal of Medical Internet Research, Journal of Medical Internet Research (Mental health), Journal of Medical Internet Research (mHealth and uHealth), Journal of Methods in Psychiatric Research, Psychiatric Rehabilitation Journal, Psychiatric Services, Psychological Assessment Schizophrenia Bulletin and Schizophrenia Research. Issues from 2009 to July 2021 were searched. These journals were chosen as they regularly published recovery-related papers.

Third, web-based searches were conducted using: Google Scholar, ResearchGate and Academia.edu. They were searched using the terms ‘experience sampling’ and ‘psychosis’, ‘ecological momentary assessment’ and ‘psychosis’, ‘experience sampling’ and ‘schizophrenia’ and ecological momentary assessment and ‘schizophrenia’. Due to the large number of results found on Google Scholar only the first five pages (100 results) per search string were searched.

Fourth, grey literature searches were conducted using OpenGrey. This was conducted using the same search terms used for the web-based searches.

Fifth, reference lists of included papers were hand-searched. Backward citation tracking was conducted by hand-searching the reference lists of all included papers. Forward citation tracking of papers citing included studies was conducted using Scopus and Google Scholar.

Finally, a panel of five experts with expertise in experience sampling methods was consulted for additional studies meeting the inclusion criteria.

## Data extraction and appraisal

Eligible citations were collated and uploaded to EndNote, and duplicates were removed. The titles of all identified citations were screened for relevance against the inclusion criteria by ED and FN, who rated all of the studies for inclusion. Data were extracted into an Excel spreadsheet developed as a Data Abstraction Table (DAT) for the review. The complete DAT can be found in (Additional file 2). Full text was obtained for potentially relevant papers and eligibility decided by the lead author.

## Quality assessment

In the absence of a typology for reporting of ESM studies, recommended reporting criteria for ambulatory studies [22] were used. Studies were assigned points based on whether they had reported elements of the study design recommended by the guidelines. Examples of recommended reporting criteria include ‘explanation of the rationale for the sampling design’ and ‘full description of the hardware and software used to collect data’. Corresponding to the number of items on the reporting criterion, the maximum possible score was 12 points. Studies scoring 0 to 6 were arbitrarily considered to be low quality, and studies scoring 7 to 12 were considered high quality. This rating was carried out by ED.

Subgroup analysis was undertaken for studies included in Objective 2 (predictors of data completeness). Subgroup analysis was not undertaken for the Objective 1 typology because the aim was to develop an exhaustive typology [23].

## Data analysis

To meet Objective 1 (design typology), design decisions were iteratively identified from the included papers. A preliminary typology of design decisions was developed by analysts who were familiar with the field of ESM (ED, MC, MS). This preliminary typology was used as headings in the initial version of the Data Abstraction Table (DAT). The constant comparative method [21] was used to refine the preliminary typology, by combining inductive category coding with simultaneous comparison of incidents observed [24]. Included papers were coded using existing DAT headings, and further or combined categories were iteratively identified [25]. The DAT was then structured using all identified design decisions and corresponding data extracted from each study [26]. Extending and combining of the preliminary typology was achieved through discussion amongst researchers ED, MC, FN, MS.

To meet objective 2, the outcome of data completeness was defined as the percentage of questionnaires completed by participants in each study out of a possible total allowed by each study protocol. This was taken directly from the paper where possible. Where percentage of data completeness was not reported, completeness was calculated by converting the total questionnaires completed during the study into a percentage using the total possible questionnaires allowed by the study protocol. The percentage represents the total data completeness for each study. Each study had a different number of questionnaires to report and different number of participants The percentage therefore includes variation between participants and within participants as questionnaire completion was completed over time. Each of the categories from the typology were used as predictor variables. Additional predictor variables included in the analysis were mean age of study participants and percentage of male participants.

A weighted regression was carried out. This approach assumes that the completeness is a summary statistic from a study with unknown variance and this standard error. Each completeness percentage statistic is weighted by how many participants were in each study. The number of questionnaires (denominator variable) was also included as a predictor to see if this predicted completeness. The completion outcome is analysed as a standard regression but where each estimate of completeness is given weight dependent on its sample size.

Predictor variables were entered into the weighted regression model. Design features not used in any included study, such as 2.1.1 ESM protocol: signal contingent, were excluded. For continuous predictor variables, cut-points were used to produce broadly equal sized categories: Participant gender (0%—32% male, 33%-65% male, 66%-100% male). For each predictor variable, the first category was then used as the reference category and each of the other categories individually and where relevant in grouped combinations were compared with the reference.

A *p*-value for each predictor was calculated using the ANOVA function comparing the difference in model fit (*R* squared value) for each predictor and the intercept model (i.e. no variables). Significant predictors were then further explored by comparing beta values in their models with their corresponding p-values. These were reported and tabulated to see the impact on completeness and to explore which differences between categories of the predictor were significantly associated with completeness.

## Results

**Fig. 1:**
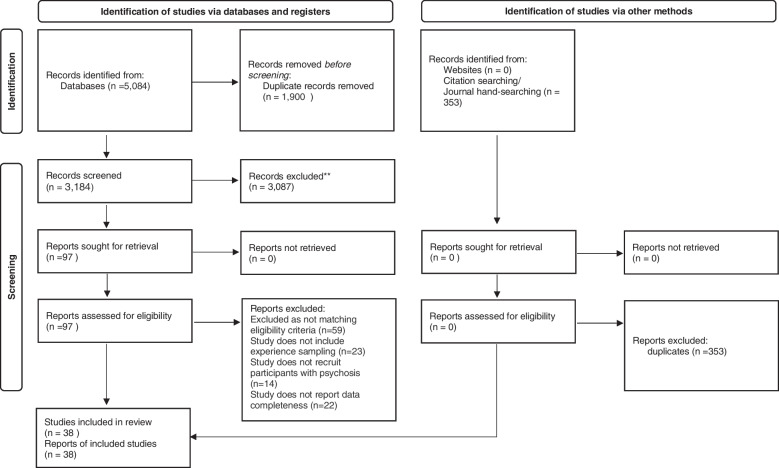
Flowchart of study selection

Thirty-eight publications were included in the review. The study selection process is summarised in Fig. 1 using the PRISMA flowchart [27].

Characteristics of included publications are presented in Table 1.

**Table 1:** Summary of included papers (*n* = 38)

High quality studies							
**#**	**Ref**	**Sample size**	**Length of data collection**	**Time period per day**	**Notification frequency**	**Hardware used**	**Data completeness**
		n	Days	Hours	n per day		%
1	[28]	24	7	13	6	PDA	88%
2	[10]	24	7	Not reported	6	PDA	97.7%
3	[29]	130	7	12	4	PDA	130/190 participants completed 2 full days or more
5	[30]	26	14	12	7	PDA	60%
11	[31]	*N* = 150 51 First episode psychosis 46 at risk mental state 53 controls	6	Not reported	10	Data collection platform	51/59 particpants completed more than 20 valid responses
13	[32]	*N* = 150 51 First episode psychosis, 46 At risk mental state, 53 controls	6	Not reported	10	Data collection platform	90.9%
14	[33]	65	28	12	4	PDA	28%
15	[34]	*N* = 49 22 schizophrenia, 27 controls	7	Personalised to each participant	5	Smartphone application	87%
18	[35]	24	6	12	4	Smartphone application	69%
25	[36]	171	7	12	7	Smartphone application	85%
31	[37]	34	6	12	10	Smartphone application	72
33	[38]	97	10	15	6	Electronic device	97 participants completed > 20 questionnaires
34	[39]	47	6	Varied	10	Smartphone application	41 participants completed at least 18 out of 60 questionnaires
38	[40]	95	6	14.5	10	Electronic device	56%
**Low quality studies**							
4	[41]	145	7		4	PDA	72.10%
6	[42]	24	7	13	6	PDA	98.10%
7	[43]	32	6	15	10	PDA	27 participants completed more than half of questionnaires
8	[44]	31	2	12	10	PDA	81%
9	[45]	53 schizophrenia, 58 controls	6	13.5	7	Data collection platform	72.10
10	[46]	22	7	Personalised for each participant	5	PDA	77.40%
12	[47]	*N* = 40	3	12	10	PDA	89.80%
16	[48]	59	5	Not reported	10	Data collection platform	98.4%
17	[49]	199	7	12	4	PDA	72.10%
19	[50]	31	7	9	4	PDA	80%
20	[51]	20	1.5	12	10	PDA	79%
21	[52]	73	1.5	12	10	PDA	74%
22	[53]	76	6	13.5		Data collection platform	71%
23	[54]	15	10	10	4	Smartphone application	76%
24	[55]	56	6	12	4	PDA	90.2%
26	[56]	141	7	12	4	PDA	69%
27	[57]	31	9	Varied	10	Smartphone application	88.5% provided ⩾30 valid responses
28	[58]	173	30	Varied	3	Smartphone application	80
29	[59]	64	21	21	6	Smartphone application	61.5
30	[60]	384	30	12	4	Smartphone application	60
32	[61]	173	30	12	3	Smartphone application	76.5
35	[62]	71	6	Not reported	10	Smartphone application	71/80 completed > 10% questionnaires
36	[63]	110	6	15	10	Electronic device	13 participants excluded as less than 20 responses completed
37	[64]	100	7	12	7	Smartphone application	85%

### Quality assessment

All 38 studies were assessed for quality. Overall, 14 (37%) were evaluated as high quality and 24 (63%) as low quality.

### Participants

The 38 included studies recruited a total of 2,722 participants with psychosis. Overall, 51% (*n* = 1,380) of participants were male. The mean age was 41 years. Other participant demographic variables were reported inconsistently across studies. Data were collected from 2,643 (97%) participants in the community and 79 (3%) were inpatients at the time of data collection. Participants had diagnoses including schizophrenia, spectrum disorder, psychosis, non-affective psychotic disorder, bipolar disorder, schizophreniform disorder, schizo-affective disorder, delusional disorder, or psychotic disorder not otherwise specified (NOS), depression with psychotic symptoms, delusional disorder, first episode psychosis and major depression.

### ESM design choices

Design choices are summarised in Table 2. Not all design decisions were reported across all studies.

**Table 2:** Design decisions used in included studies (*n* = 38)

Design decision	n
**Study design**	
Cohort study	19
Case control study	17
Crossover trial	1
Randomized repeated measures crossover design	1
**Sample size**	
Mean	73.6
SD	74.7
**Data collection setting**	
Hospital	3
Community	34
**Recruitment setting**	
Hospital	3
Community	30
Hospital and community	2
**ESM Protocol**	
Event-contingent	0
Signal-contingent assessments	27
Time-contingent assessments	7
Stratified	3
**Type of prompt**	
Auditory (beep)	2
Vibrate	25
Auditory (beep) and vibrate	0
**Technology used**	
Personal Digital Assistant (PDA)	16
Smartphone application	14
Data collection platform	5
Electronic device	3
**Device ownership**	
Provided by researcher	34
Participants provided with device if they didn’t have one	2
**Time taken to complete measures (minutes)**	
Mean	3.5
SD	1.22
**Type of data being collected**	
Experience data	12
Context data	0
Behavioural data	1
Cognitive data	1
Experience and context data	11
Experience and behavioural data	6
Experience, context and behavioural data	0
Behavioural and context data	2
Experience, context, behavioural and cognitive data	0
**Design of the questionnaire**	
Derived from validated scale/scales	6
Uses non validated scales	19
Combination of both	8
**Other data collected**	
Yes	9
No	29
**Total length of time per day sampling took place (hours)**	
Mean	12.3
SD	1.3
**Questionnaire availability (minutes)**	
Mean	16.4
SD	13.2
**Data collection frequency (Number of prompts per day)**	
Mean	6.8
SD	2.7
**Study duration (days)**	
Mean	9.3
SD	7.8
**Terminology used**	
Experience Sampling Methods or ESM	23
Ecological Momentary Assessment or EMA	12
Ambulatory assessment	0
EMAc	1
Mobile phone based assessment	1
Self assessment	0
Computerised ambulatory monitoring	1
**Data access**	
Researchers	37
Participants	0
Researchers, Participants and clinicians	1
**Accepted response rate (%)**	
Mean	27.8
SD	9.0
**Contact with research team during data collection**	
Researcher initiated	16
Participant initiated	1
Both	5
**Participant training**	
Yes	33
No	0
**Additional support**	
Initial contact after commencing data collection	5
Initial contact after commencing data collection + technical support on request	5
Initial contact after commencing data collection + additional support on request	1
Technical support on request	3
Technical support on request + motivational support + emotional support	1
Motivational support + technical support	1
Initial contact after commencing data collection + motivational support	2
Motivational support	4
Initial contact after commencing data collection + technical support + motivational support	1
**Participant reimbursement (£)**	
Mean	60.7
SD	38.7
**Data completeness %**	
Mean	76.8
SD	15.2

### Data completeness

**Fig. 2:**
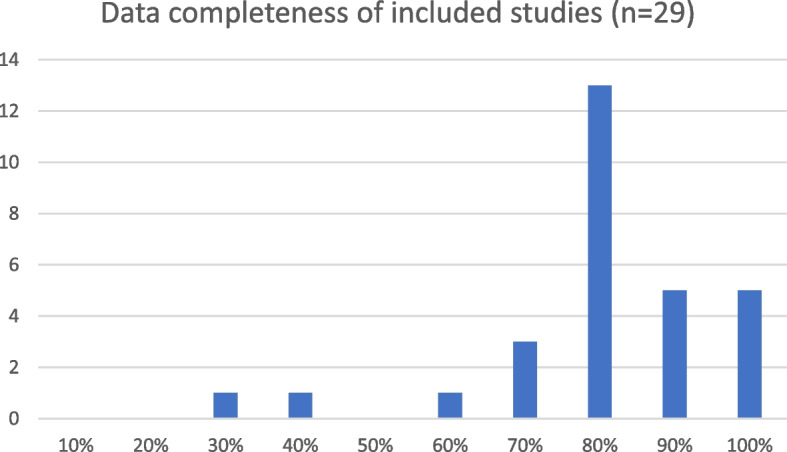
Data completeness of included studies

Percentage of data completeness was obtained for 29 studies. The remaining nine studies expressed data completeness as either a percentage or number of participants who completed more than a predefined threshold amount, meaning it was not possible to determine the exact data completeness percentage. Data completeness across included studies in summarised in Fig. 2.

#### Objective 1: Typology of design choices used in ESM studies

Analysis of included publications identified 24 design decisions. Three superordinate themes were identified from the designs: Study context, ESM approach and ESM implementation.

##### Superordinate theme 1: Study context

The Study Context theme describes decisions made when designing an ESM study which are not ESM-specific decision. These are shown in Table 3.

**Table 3:** Superordinate theme 1: Study context

Theme	Definition	Categories
1.1 Study design	Design of the overall study within which the ESM study is nested	Cohort
		Case control
		Crossover trial
		Pilot RCT
1.2 Sample size	Intended number of participants completing ESM measures	N
1.3 Data collection setting	Location of ESM data collection	Hospital (currently an in-patient)
		Community (not an in-patient)
1.4 Recruitment setting	Location of participant recruitment	Hospital
		Community
		Hospital and community

##### Superordinate theme 2: ESM Approach

ESM approach describes the design decisions relating specifically to experience sampling and are shown in Table 4.

**Table 4:** Superordinate theme 2: ESM Approach

Theme	Definition	Categories
**2.1 Data collection method**		
2.1.1 ESM protocol	Data collection trigger	Event-contingent assessments: carried out when a predefined event has occurred, for example attending a social event
		Signal-contingent assessments: scheduled at random times
		Time-contingent assessments: involve prompting the individual to make an assessment at pre-defined intervals
		Hybrid assessments: a combination of more than one data collection trigger
2.1.2 Type of prompt	Alert to promote participant response	Auditory (beep)
		Vibrate
		Visual prompt
2.1.3 Hardware used	The hardware used to collect data	Personal Digital Assistant (PDA)
		Online data collection platform
		Smartphone
2.1.4 Software used	The software used to collect data	Smartphone application
2.1.5 Device ownership	Owner of the device used to collect data	Provided by researcher
		Pre-owned by participant
**2.2 Measures**		
2.2.1 Questionnaire completion time	Total time taken by participants to complete one round of questions	minutes (n)
2.2.2 Type of data	Type of data being collected	Cognitive data (e.g., Mobile word task)
		Behavioural data (e.g., instance of cannabis use)
		Experience data (e.g., current mood)
		Context data (e.g., immediate physical environment)
2.2.3 Questionnaire design	Were questions derived from psychometrically validated scales?	Derived from validated scale/scales
		Uses non-validated scales
2.2.4 Other data collected?	Was other participant data collected during the ESM data collection period?	Yes
		No
**2.3 Schedule**		
2.3.1 Measurement duration	Time period within each day that data collection takes place, i.e., gap between earliest and latest time	Hours (n)
2.3.2 Questionnaire availability	How long was the questionnaire available for participant response after each prompt?	Minutes (n)
2.3.3 Data collection frequency	Number of prompts per day	n
2.3.4 Length of time in study	Length of time ESM data collection is conducted	Days (n)

##### Superordinate theme 3: ESM Implementation

The theme of ESM implementation is shown in Table 5.

**Table 5:** Superordinate theme 3: ESM Implementation

Theme	Definition		Categories
**3.1 Terminology**		What was the method of data collection called in the study?	Experience Sampling Methods or ESM
			Ecological Momentary Assessment or EMA
			Ambulatory assessment
			Computerised ambulatory monitoring
			Mobile phone-based assessment
			Self-assessment
			Computerised ecological momentary assessment (EMAc)
**3.2 Data**			
3.2.1 Data access		Who can access collected ESM data?	Researchers
			Participants
			Clinicians
3.2.2 Accepted response rate		Amount of questionnaires completed in order to be eligible for inclusion	Continuous: %
**3.3 Participation**			
3.3.1 Participant training?		Was training provided for participants prior to commencing ESM data collection?	Yes
			No
3.3.2 Contact with research team		Who initiates additional contact between the research team and participants during the ESM period?	Researcher Participant
			Either
3.3.3 Additional support		Support offered to participants by the research team after commencing data collection	None
			Initial contact after commencing data collection
			Technical support
			Technical support on request
			Motivational support
			Emotional support
3.3.4 Participant reimbursement		Compensation provided to each participant	Continuous: £

#### Objective 2: Predictors of data completeness

A weighted regression of design decisions included in the typology was conducted, and the significance of each design choice as a predictor of data completeness is shown in Table 6.

**Table 6:** Weighted regression of design choices as predictors of data completeness (29 studies)

**Design Choice**	**Significance** (overall *p*-value)
1.1 Study design	0.221
1.2 Sample size	0.159
1.3 Data collection setting	0.737
1.4 Recruitment setting	0.741
**2.1.1 ESM protocol**	**0.021**
2.1.2 Type of prompt	0.310
2.1.3 Hardware used	0.078
2.2.2 Type of data	0.978
2.2.3 Questionnaire design	0.540
**2.2.4 Other data collected?**	** < 0.001**
**2.3.1 Measurement duration**	**0.033**
2.3.2 Questionnaire availability	0.210
**2.3.4 Length of time in study**	**0.021**
**3.2.2 Accepted response rate**	**0.035**
**3.3.2 Contact with research team**	**0.014**
3.3.3 Additional support	0.390
Participant gender	0.435
Participant mean age	0.450
Number of questionnaires	0.165
2.1.5 Device ownership	0.320
3.1 Terminology used	0.984
3.2.1 Data access	0.869
3.3.4 Participant reimbursement	0.313

Bold =*p* < 0.05

The regression identified six candidate predictors of data completeness: ESM protocol, length of time per measurement, total time in the study, research team contact, accepted response rate and collecting other data. The findings from the weighted regression for specific values of these six candidate predictors are shown in Table 7.

**Table 7:** Weighted regression for candidate predictors of data completeness (29 studies)

Design Choice	Beta-value	Standard error	p
2.1.1 Protocol			
Signal contingent	-11.82		
Time contingent vs Signal contingent	-0.25	4.24	**0.010**
Hybrid vs signal contingent		5.18	0.962
2.2.4 Other data collected			
Yes	19.26		
No vs yes		3.57	** < 0.001**
2.3.1 Measurement duration (hours)	-2.16	0.94	**0.033**
2.3.4 Length of time in study (days)	-0.438	0.18	**0.021**
3.2.2 Accepted response rate (% of questionnaires)	68.07	28.70	**0.035**
3.3.2 Contact with research team			
Researcher initiated	-17.49		
Participant initiated vs Researcher initiated	-4.84	5.06	**0.004**
Both vs Researcher initiated		7.85	0.549

Table 7 shows that using a time contingent protocol rather than a signal contingent protocol was significantly associated with reduced data completeness by around 12%. Greater data collection burden was consistently associated with reduced data completeness: every extra hour in measurement duration reduced data completeness by 2%, every additional day enrolled in the study reduced data completeness by 0.5%, and collecting extra data alongside ESM data reduced data completeness by 19%. Finally, researcher-initiated contact with participants increased data completeness by 17.5% when compared to participant-initiated contact.

### Sensitivity analysis

The analysis was repeated only including the 10 studies rated as high quality that expressed the data completeness as a percentage. The quality assessment ratings for studies is shown in Additional file 2. 14 studies were rated as high quality. The quality criteria met by fewest studies was justification of sample size (met by 3 studies) and rationale for the sampling design (met by 5 studies). The weighted regression identified 3 design decisions that predicted of data completeness: sample size (*p*: 0.012), other data collected (*p*: 0.006) and hardware used (*p*: 0.045). The statistically significant predictors with beta values, standard errors and *p*-values can be found in additional file 3.

## Discussion

This systematic review identified design decisions used in experience sampling studies of people with psychosis. The resulting typology identified three superordinate themes relating to design decisions in ESM studies: Study context, ESM approach and ESM implementation. Weighted regression was then used to identify six design decisions that predicted data completeness: ESM protocol, other data collected, length of time in study, measurement duration, accepted response rate and contact with the research team.

### Objective 1: Typology of design choices used in digital ESM studies

A systematic search of published literature on ESM allowed the creation of a typology that accurately represent the methods used in the field [65]. The resulting typology can help researchers to choose designs, help establish a common language and help to provide the field of ESM research with organisational structure [66].

Four ESM protocols were included in the typology. Event-contingent assessments, Signal-contingent assessments, Time-contingent assessments, and hybrid assessments. Three ESM protocols are commonly cited in ESM literature [67]. A questionnaire prompt may be sent to participants at pre-defined intervals (time contingent), scheduled at random times (signal contingent) or carried out when a predefined event has occurred (event contingent) [17]. ‘Hybrid assessment’ has been used to describe combined protocols.

A sampling protocol is often selected based on the variables of interest [16]. Choice of protocol may depend on whether the variables are discrete, relating to distinct events such as social interactions, or continuous events with less identifiable parameters, such as mood [4]. Discrete events are well suited to event contingent protocols as they have definable beginning and end points. Rather than waiting for a signal or prompt, participants fill out a questionnaire when a discrete event occurs. Time contingent and signal contingent protocols are better suited to measurement of continuous variables. Participants are not required to identify the beginning or end of a pre-defined event in order to complete a questionnaire in time contingent or signal contingent designs [9].

Signal and time contingent protocols can be carried out at fixed or flexible time points [9]. Some authors described their signal contingent designs as stratified [37, 39, 43] or semi-random [16]. In stratified sampling, questionnaires are sent at random time points within pre-programmed time windows. These parameters are unknown to the participant [58]. An example of this is a protocol with a range of 90 min within which at least one beep occurred with a minimum of 15 min and a maximum of 3 h between each beep. The intention of the stratified protocol is to balance the requirement for collecting variable and valid data with participant burden [43]. The data may be more variable than a time contingent protocol, as the timing of the signal cannot be anticipated by participants. Participants will not therefore be less likely to alter their daily life or habits to incorporate the sampling. Some stratified sampling protocols included personalising the daily measurement period to each participant’s waking hours [39, 58].

Another design choice relating to ESM approach is whether the digital technology used for data collection was provided by the researchers or participants were required to use their own smartphones. The present review found there to be no significant difference in data completeness between studies which provided a device and those in which participants used their own phone. There is conflicting opinion about this in ESM literature [22]. Disadvantages of using participant-owned devices may include increased distractions from other applications on the phone and decreased uniformity of study procedures [68]. Advantages may include reduced study costs and also reduced requirement for participants to meet researchers face to face [69], which could reduce participant burden. A meta-analysis on ESM protocol compliance in substance users found no significant difference in adherence rates for participants who used their own phone compared with participants who used researcher provided devices [70].

The typology identified measures used for ESM studies that were derived from psychometrically validated scales and others that were not validated. Many of the measures which were not validated were created by the research team for the purpose of the study. In ESM studies, researchers have often selected items from longer, validated measures and adapted the questions to fit the study time frame [22]. This is often due to the lack of validated measures available for use in ESM studies [71]. Researchers should consider that adding “right now” to a questionnaire item does not necessarily mean that it is appropriate for measuring momentary states [3]. Measuring momentary experiences is different from measuring phenomenon included in cross-sectional questionnaires that occur generally and retrospectively [9]. When considering what questionnaires to use in ESM studies, researchers should take into account the momentary nature of the phenomena and develop items that accurately capture how they are experienced over the course of the study duration [72].

The typology also identified support offered to participants once data collection has commenced. This is a common method of encouraging protocol adherence [13]. It can take the form of technical support, motivational support, or emotional support.

This study found no significant association between data completeness and reimbursement to participants. However, reimbursement can involve a number of different strategies, including providing added incentives to participants who achieve high levels of protocol adherence, withholding payment if compliance falls below a certain threshold, and providing payment at regular face to face meetings [22]. The value of participant reimbursement has been found to be positively associated with protocol adherence [15]. However, the authors note that they did not consider the strategy used to provide the incentives. Instead, a total incentive was calculated for each study. Another study investigated studies which provided reimbursement proportional to the number of questionnaires completed. No increase in protocol adherence was found [70]. The difference in findings indicate that more research is needed in this area, particularly on the influence of different reimbursement strategies on data completeness.

#### Applicability to different populations

Design choices included in the typology are consistent with those described in suggested ESM reporting guidelines for research in psychopathology [22]. This suggests that the typology can be applied across different mental health populations. Future research is required to validate the typology for use with transdiagnostic groups. For example, when being used to collect data from participants with depression, or measuring discrete variables such as self-harming behaviours [73]. This suggests that event contingent protocols may be used more frequently with this population [4].

### Objective 2: Design decisions that predict data completeness

The ESM protocol used predicted data completeness. Using a signal contingent protocol compared to a time contingent protocol was shown to increase data completeness by around 12%. This is in contrast with previous research which has shown that signal contingent sampling may be perceived as more burdensome by study participants [74] leading to lower levels of adherence compared to other protocols [75]. The authors suggest that higher levels of predictability afforded by time contingent protocols may increase adherence as participants are able to integrate responding to questionnaires into their daily routine [75]. Knowledge of when to expect the questionnaire prompts may allow participants to plan their daily tasks in accordance with the scheduled questionnaires [15].

A study of ESM in participants with substance dependence found that participants may prefer to isolate themselves, or to be in quiet environment when responding to questionnaires [76]. In this case, the additional burden of anticipating the signal at a certain time and finding a quiet environment may account for lower data completeness with a time contingent protocol. Similarly, the psychosis population in our review may have more cognitive impairments such as reduced attention, meaning that the potential for integrating data collection into daily life is reduced, so a signal contingent assessment is easier to provide an immediate response to. As there are advantages and disadvantages to each ESM protocol and inconsistent findings regarding their effects on data completeness, it has been suggested that the choice of protocol should be based on the requirements of the study [15]. This may involve choosing a protocol that is based on the nature of the variables of interest [4].

Design decisions relating to scheduling were found to influence data completeness. Longer study lengths and longer daily measurement duration predicted lower levels of data completeness. For every day participants were enrolled in a study, data completeness reduced by 0.5%. Similarly, every additional hour of measurement duration per day reduced data completeness by 2%. These findings are consistent with previous research. A study analysed predictors of adherence to ESM protocols in a pooled data set of 10 ESM studies. The sample consisted of 1,717 participants, of whom 15% had experienced psychosis. The results showed that ESM protocol adherence declined over the duration of study days [13]. More generally, the problems experienced by people living with psychosis, such as negative symptoms and amotivaton, may require lower burden data collection procedures.

Some studies have customised the time period per day that sampling took place for each participant. This has included personalising the daily measurement period to each participant’s waking hours [29, 34, 36]. Sampling took place for the same number of hours per day for each participant but began and ended at different times. This review only included the total number of hours per day sampling took place in the analysis. Future research could investigate the relationship between personalised scheduling and data completeness. Completeness, to allow diurnal variation in symptomatology to be integrated into the data collection schedule. For example, an individual who is more preoccupied with hallucinatroy experiences in the morning may be more able to respond to data collection prompts later in the day.

Monitoring participants once ESM has commenced has been recommended in order to encourage protocol adherence [22]. Support from researchers during the data collection phase is either initiated by researchers or by participants. Researcher initiated contact with participants throughout the duration of the study increased data completeness by 17.5% compared to participant-initiated contact. These findings support active researcher support once data collection has commenced. This is something which may be particularly beneficial if participants find the study procedures burdensome.

## Strengths and limitations

One strength of this study is the rigorous search strategy. This was designed in collaboration with two information specialists with expertise in conducting systematic review searches in the field of mental health. Another strength is the use of several analysists with differing expertise. Areas of expertise include clinical expertise, mental health services research and technology research and design.

Several limitations can be identified. Studies were only included which reported data completeness, or studies where it was possible to calculate this. Studies that did not report this could have been included for Objective 1, which may have increased generalisability of the findings. Similarly, studies were only included if they used digital ESM which may also have limited generalisability. The title, abstract and full paper sifting was only carried out by one author (ED), which may introduce inclusion bias. In the absence of an appropriate quality checking tool, recommended reporting guidelines were used instead, which may not have been fully capturing quality. Finally, a meta regression could not be carried out, and the weighted regression that was conducted instead does not account for given estimates of variance, meaning that conclusions drawn from the analysis need to be interpreted with caution. Additionally, a small number of studies (*n* = 10) were included in the sensitivity analysis.

## Conclusions

The study addresses a knowledge gap related to design decisions for ESM studies recruiting people with psychosis. The typology of design choices used in ESM studies identifies key design decisions to consider when designing and implementing an experience sampling studies. The typology could be used to inform the design of future experience sampling studies in transdiagnostic mental health populations. The review also identifies a number of predictors of data completeness. This knowledge could help future researchers to increase the likelihood of achieving fuller data sets.

Future research might seek to add additional design choices to the typology and to refine design decisions as the field advances. Future research may also examine how the typology is used by researchers when designing ESM studies. Researchers may also validate the typology for use with different mental health populations.

## Supplementary Information

**Additional file 1.** Sources, search strategy, and study selection.

**Additional file 2.** Data abstraction table.

**Additional file 3:** **Additional Table 1.** Overall p-values for predictors of percentage completeness for high quality studies only (*n*=10). **Additional Table 2.** Statistical significant predictors with beta values, standard errors and *p*-values, for high quality studies (*n*=10).

**Additional file 4.** Quality assessment.

## Data Availability

All data generated or analysed during this study are included in this published article and its supplementary information files.

## Abbreviations

ESM: Experience Sampling Method
DAT: Data abstraction table
