# Supplementary material for: Design decisions and data completeness for experience sampling methods used in psychosis: systematic review
Source: BMC Psychiatry. 2022 Oct 28;22:669. doi: 10.1186/s12888-022-04319-x (PMC9617456; doi:10.1186/s12888-022-04319-x)
Supplement: Supplementary file 1 — Additional file 1. Sources, search strategy, and study selection. [file 12888_2022_4319_MOESM1_ESM.docx]

## Sources, Search Strategy, and Study Selection

The search for papers was performed using six sources.

Electronic databases (n=5) were searched: MEDLINE (Ovid), EMBASE, PsycINFO, Cochrane Library and Web of Science. The following combinations of search terms were used:

1 exp ecological momentary assessment

2 exp sampling

3 exp ambulatory monitoring/ or exp telemonitoring

4 exp self evaluation

5 ("experience sampl*" or "sampling method*").tw,kw.

6 ("momentary assessment*" or ESM or EMA).tw,kw.

7 "momentary intervention*".tw,kw.

8 (data adj2 (captur* or collect* or real?time or "time series" or acqui* or obtain* or gather* or entry or entries or input*)).tw,kw.

9 ("daily diary method " or "daily diary stud*" or track* or monitor* or assess* or measur* or sensing or sense or "self?report*").tw,kw.

10 or/1-9

11 technology

12 software

13 exp mobile application

14 exp mobile phone

15 exp telemedicine

16 *sensor

17 exp text messaging

18 (Telemedicine or Smartphone* or smart-phone* or "cell phone*" or "mobile phone" or App or Apps or "Mobile application*" or Mhealth or m-health or ehealth or e-health or wearable* or remote or "Personal digital assistant*" or Software or "mobile technolog*" or "mobile device*" or "mobile electronic device*" or "cellular telephone*" or "cellular phone*").tw,kw.

19 or/11-18

20 10 and 19

21 exp psychosis

22 exp schizophrenia

23 (psychoses or psychosis or psychotic or schizo*).tw,kw.

24 or/21-23

25 20 and 24

26 limit 25 to English language

Keyword, and where applicable subject headings, were tailored to each database. All databases were searched from 1^st^ January 2009- June 2021

The search terms were modified for each database. For example, MeSH terms used for experience sampling in the Cochrane library included ‘Monitoring, Ambulatory’ rather than two separate terms ‘Monitoring’ OR ‘Ambulatory’ used in EMBASE. Also, the Cochrane library used the MeSH term ‘Wearable Electronic Devices’ which was ‘Wearable Devices’ in PsycINFO.

The many different terms used in the literature to describe experience sampling methods required broader terms associated more generally with data collection, such as ‘diary study’, ‘input’ monitor’ or ‘measure’, which are not generally associated with technology.

Also ‘sensing’ is associated with technology used in passive data collection, but a number of studies use active and passive data collection alongside each other or used the term sensing more generally to refer to the active or passive use of mobile sensors
