# Supplementary material for: Design decisions and data completeness for experience sampling methods used in psychosis: systematic review
Source: BMC Psychiatry. 2022 Oct 28;22:669. doi: 10.1186/s12888-022-04319-x (PMC9617456; doi:10.1186/s12888-022-04319-x)
Supplement: Supplementary file 3 — Additional file 3: Additional Table 1. Overall p-values for predictors of percentage completeness for high quality studies only (n=10). Additional Table 2. Statistical significant predictors with beta values, standard errors and p-values, for high quality studies (n=10). [file 12888_2022_4319_MOESM3_ESM.docx]

**Additional Table 1. Overall p-values for predictors of percentage completeness for high quality studies only (n=10)**

| **Design Choice** | **Significance**  **(overall p-value)** |
| --- | --- |
| 1.1 Study design | 0.234 |
| 1.2 Sample size | **0.012** |
| 1.3 Data collection setting | NA  (all community) |
| 1.4 Recruitment setting | NA  (all community) |
| 2.1.1 Protocol | 0.697 |
| 2.3.3 Data collection Frequency | 0.589 |
| 2.2.2 Type of data | 0.204 |
| 2.2.3 Questionnaire design | 0.453 |
| 2.2.4 Other data collected | **0.006** |
| 2.3.2 Questionnaire availability | 0.119 |
| 2.3.4 Length of time in study | 0.595 |
| 2.3.1 Measurement duration | 0.133 |
| 3.2.2 Accepted response rate | 0.506 |
| 3.3.2 Contact with research team | 0.669 |
| 3.3.3 Additional support | 0.693 |
| % male | 0.331 |
| Mean age | 0.636 |
| 2.1.2 Type of prompt | NA  (only one category) |
| 2.1.3 Hardware used | **0.045** |
| 2.1.5 Device ownership | NA  (only one category) |
| 3.1 Terminology used | 0.735 |
| 3.2.1 Data access | 0.675 |
| 3.3.4 Participant reimbursement | 0.935 |

Bold = p<0.05

**Additional Table 2. Statistical significant predictors with beta values, standard errors and p-values, for high quality studies (n=10).**

| **Design Choice** | **Beta-value** | **Standard error** | **p** | **Overall p-value** |
| --- | --- | --- | --- | --- |
| **Sample size**  0-49 participants  50-99 participants vs 0-49 participants  100+ participants vs 0-49 participants | REF  -23.55  8.20 | 8.02  5.74 | 0.022  0.196 | **0.0125** |
| **Other data collected**  Yes  No vs yes | REF  28.47 | 7.71 | 0.006 | **0.006** |
| **Hardware used**  Personal Digital Assistant  Data collection platform vs Personal Digital Assistant  Smartphone application Personal Digital Assistant  Electronic device Personal Digital Assistant | REF  10.00  1.49  -24.90 | 8.86  7.96  9.87 | 0.302  0.858  0.045 | **0.045** |
